# Inflammation and olfactory loss are associated with at least 139 medical conditions

**DOI:** 10.3389/fnmol.2024.1455418

**Published:** 2024-10-11

**Authors:** Michael Leon, Emily T. Troscianko, Cynthia C. Woo

**Affiliations:** ^1^Department of Neurobiology and Behavior, University of California, Irvine, Irvine, CA, United States; ^2^Center for the Neurobiology of Learning and Memory, University of California, Irvine, Irvine, CA, United States; ^3^Institute for Memory Impairments and Neurological Disorders, University of California, Irvine, Irvine, CA, United States; ^4^The Oxford Research Centre in the Humanities, University of Oxford, Oxford, United Kingdom

**Keywords:** olfaction, inflammation, medical conditions, causation, correlation, olfactory dysfunction, olfactory enrichment

## Abstract

Olfactory loss accompanies at least 139 neurological, somatic, and congenital/hereditary conditions. This observation leads to the question of whether these associations are correlations or whether they are ever causal. Temporal precedence and prospective predictive power suggest that olfactory loss is causally implicated in many medical conditions. The causal relationship between olfaction with memory dysfunction deserves particular attention because this sensory system has the only direct projection to memory centers. Mechanisms that may underlie the connections between medical conditions and olfactory loss include inflammation as well as neuroanatomical and environmental factors, and all 139 of the medical conditions listed here are also associated with inflammation. Olfactory enrichment shows efficacy for both prevention and treatment, potentially mediated by decreasing inflammation.

## 1 Introduction

### 1.1 Observations linking olfactory loss and medical conditions: correlation, precedence, and prediction

#### 1.1.1 Olfactory loss is associated with many medical conditions

First, a strikingly large number of medical conditions are accompanied by olfactory dysfunction (Tables 1–3). The remarkably long and diverse list of medical conditions that co-occur with olfactory loss raises the possibility that there is something deeper to these relationships.

**Table 1:** Neurological condition/disorder, the reference for accompanying olfactory dysfunction, study size of olfactory study, and reference for inflammation.

**Medical condition**	**Olfactory dysfunction**	**Olfactory study size (*N*)**	**Inflammation**
Agnosia (olfactory)	Kopala and Clark, 1990	77	Wang et al., 2010
Alcoholism	Rupp et al., 2004	60	Leclercq et al., 2017
Alzheimer's disease	Waldton, 1974	100	Xie et al., 2022
Amyotrophic lateral sclerosis	Viguera et al., 2018	147	McCombe and Henderson, 2011
Anesthesia cognitive impairment	Zhang C. et al., 2022	242	Subramaniyan and Terrando, 2019
Anorexia nervosa	Roessner et al., 2005	32	Dalton et al., 2018
Anxiety	Chen X. et al., 2021	107	Guo B. et al., 2023
Autism	Kinnaird et al., 2020	80	Kern et al., 2016
Cerebral palsy	Nakashima et al., 2019	14	Paton et al., 2022
Cervical dystonia	Marek et al., 2018	198	Scorr et al., 2024
Childhood maltreatment	Croy et al., 2010	22	Wong et al., 2022
Cluster headache	Samanci et al., 2021	57	Hardebo, 1994
Corticobasal syndrome	Luzzi et al., 2007	7	Alster et al., 2021
Creutzfeldt-Jakob disease	Reuber et al., 2001	1	López González et al., 2016
Depression (unipolar)	Eliyan et al., 2021	3,546	Kofod et al., 2022
Depression (bipolar)	Kazour et al., 2020	176	Benedetti et al., 2020
Epilepsy	Khurshid et al., 2019	912	Rana and Musto, 2018
Essential tremor	Elhassanien et al., 2021	46	Muruzheva et al., 2022
Fibromyalgia	Amital et al., 2014	45	Coskun Benlidayi, 2019
Frontotemporal dementia	Luzzi et al., 2007	11	Bright et al., 2019
Glioblastoma	Kebir et al., 2020	122	Zhang H. et al., 2022
Gulf war illness	Chao, 2024	80	Michalovicz et al., 2020
Headache	Gossrau et al., 2023	80	Biscetti et al., 2021
Heavy metal exposure	Renzetti et al., 2024	130	He et al., 2024
Hepatic encephalopathy	Zucco et al., 2006	24	Lu, 2023
Herpetic meningoencephalitis	Landis et al., 2010	3	Li et al., 2023
Huntington's disease	Fernandez-Ruiz et al., 2003	162	Valadão et al., 2020
Idiopathic intracranial hypertension	Bershad et al., 2014	38	Sinclair et al., 2008
Impulsive violent offenders	Challakere Ramaswamy et al., 2023	485	Hasan Balcioglu et al., 2022
Lewy body dementia	Yoo et al., 2018	217	Amin et al., 2022
Loneliness	Desiato et al., 2021	221	Van Bogart et al., 2022
Long COVID-19	Burges Watson et al., 2021	9,000	Aiyegbusi et al., 2021
ME/chronic fatigue syndrome	Harris et al., 2017	11	Chaves-Filho et al., 2023
Memory loss with aging	Doty et al., 1984	1,995	Sartori et al., 2012
Menopause	Lee et al., 2019	3,863	Malutan et al., 2014
Migraine headaches	Whiting et al., 2015	100	Kursun et al., 2021
Mild cognitive impairment	Peters et al., 2003	100	Leonardo and Fregni, 2023
Motor neuron disease	Hawkes et al., 1998	193	Komine and Yamanaka, 2015
Multiple sclerosis	Atalar et al., 2018	55	Groppa et al., 2021
Multiple-system atrophy	Abele et al., 2003	8	Rydbirk et al., 2022
Myasthenia gravis	Leon-Sarmiento et al., 2012	27	Koneczny and Herbst, 2019
Myotonic dystrophy	Masaoka et al., 2011	7	Azotla-Vilchis et al., 2021
Narcolepsy	Buskova et al., 2010	66	Valizadeh et al., 2024
Neuromyelitis optica	Schmidt et al., 2013	20	Kümpfel et al., 2024
Obsessive compulsive disorder	Berlin et al., 2017	30	Marazziti et al., 2023
Parkinson's disease	Haehner et al., 2009	50	Pajares et al., 2020
Posterior cortical atrophy	Witoonpanich et al., 2013	15	Firth et al., 2019
Postoperative delirium	Brown et al., 2015	165	Pang Y. et al., 2022
Postpartum depression	Peng et al., 2021	39	Bränn et al., 2020
Posttraumatic stress disorder	Vasterling et al., 2000	68	Hori and Kim, 2019
Prenatal alcohol syndrome	Bower et al., 2013	16	Masehi-Lano et al., 2023
Progressive supranuclear palsy	Shill et al., 2021	281	Alster et al., 2020
Psychopathy	Bettison et al., 2013	381	Wang et al., 2017
Psychosis	Kamath et al., 2024	195	Misiak et al., 2021
Pure autonomic failure	Goldstein and Sewell, 2009	51	Brás et al., 2020
Radioactive iodine	Suat et al., 2016	63	Stanciu et al., 2023
REM sleep behavior disorder	Iranzo et al., 2021	140	Kim et al., 2019
Repetitive head impacts	Alosco et al., 2017	123	McKee et al., 2014
Restless leg syndrome	Adler et al., 1998	46	Jiménez-Jiménez et al., 2023
Schizophrenia	Kopala et al., 1993	98	Müller, 2018
Semantic dementia	Luzzi et al., 2007	20	Pascual et al., 2021
Sexual dysfunction	Siegel et al., 2021	1,981	Yafi et al., 2016
Sociopathy	Mahmut and Stevenson, 2012	79	Wang et al., 2017
Sodium channel Nav1.7 mutation	Weiss et al., 2011	3	Cheng et al., 2021
Spinocerebellar ataxia type 7	Galvez et al., 2014	55	Goswami et al., 2022
Stroke	Wehling et al., 2015	78	Lambertsen et al., 2019
Subarachnoid hemorrhagic surgery	Bor et al., 2009	197	Hokari et al., 2020
Tinnitus	Katayama et al., 2023	510	Kang et al., 2021
Tourette syndrome	Kronenbuerger et al., 2018	56	Alshammery et al., 2022
Traumatic brain injury	Frasnelli et al., 2016	63	Postolache et al., 2020
Vascular dementia	Suh et al., 2020	1	Trares et al., 2022
Zika/Guillain-Barré syndrome	Lazarini et al., 2022	38	Acosta-Ampudia et al., 2018

**Table 2:** Somatic condition/disorder, the reference for accompanying olfactory dysfunction, study size of olfactory study, and reference for inflammation.

**Medical condition**	**Olfactory dysfunction**	**Olfactory study size (*N*)**	**Inflammation**
Adenoid hypertrophy	Konstantinidis et al., 2005	65	Ye et al., 2022
Allergic rhinitis	Apter et al., 1999	90	Klimek and Eggers, 1997
Anemia	Dinc et al., 2016	100	Weiss et al., 2019
Arthritis	Steinbach et al., 2011	101	Gwinnutt et al., 2022
Asthma	Rhyou et al., 2021	68	Gillissen and Paparoupa, 2015
Autoimmune encephalitis	Geran et al., 2019	64	Graus et al., 2016
Behcet disease	Akyol et al., 2016	96	Nair and Moots, 2017
Blepharospasm	Gamain et al., 2021	34	Lu et al., 2014
Cancer (head and neck)	Spotten et al., 2016	40	Bonomi et al., 2014
Candida infection	Fluitman et al., 2021	218	Dahlman et al., 2021
Cardiovascular disease	Roh et al., 2021	20,016	Bafei et al., 2023
Celiac disease	Berkiten et al., 2024	74	Barone et al., 2022
Chagas' disease	Leon-Sarmiento et al., 2014	120	Nunes et al., 2023
COPD	Thorstensen et al., 2022	183	Barnes, 2016
Cirrhosis	Garrett-Laster et al., 1984	45	Dirchwolf and Ruf, 2015
Congestive heart failure	Chamberlin et al., 2024	477	Cesari et al., 2003
Corticobasal syndrome	Luzzi et al., 2007	40	Alster et al., 2021
COVID-19	Vaira et al., 2020	150	Radke et al., 2024
Crohn's disease	Fischer et al., 2014	123	Petagna et al., 2020
Cushing syndrome	Heger et al., 2021	60	Wurth et al., 2022
Diabetes	Zhang et al., 2019	105	Lontchi-Yimagou et al., 2013
Erectile dysfunction	Deng et al., 2020	102	Kaya-Sezginer and Gur, 2020
Frailty	Van Regemorter et al., 2022	155	Soysal et al., 2016
Glaucoma	Iannucci et al., 2024	NS	Baudouin et al., 2021
*Helicobacter pylori* infection	Üstün Bezgin et al., 2017	66	Guo X. et al., 2023
HIV/AIDS	Zucco and Ingegneri, 2004	48	Deeks et al., 2013
Hypertension	Datta et al., 2023	60	Patrick et al., 2021
Hypothyroidism	McConnell et al., 1975	18	Kubiak et al., 2023
Idiopathic inflammatory myopathy	Iaccarino et al., 2014	120	Lundberg et al., 2021
Inflammation	Schubert et al., 2015	1,611	Schubert et al., 2015
Inflammatory bowel disease	Sollai et al., 2021	199	Shi et al., 2006
Ischemic heart failure	Akşit and Çil, 2020	80	Rao et al., 2021
Kidney disease	Frasnelli et al., 2002	64	Rayego-Mateos et al., 2023
Laryngectomy	Veyseller et al., 2012	30	Akizuki et al., 2022
Leptin imbalance	East and Wilson, 2019	NS	Likuni et al., 2008
Macular degeneration	Kar et al., 2015	138	Tan et al., 2020
Malnutrition	Gunzer, 2017	NS	Muscaritoli et al., 2023
Obesity	Velluzzi et al., 2022	80	Cox et al., 2015
Obstructive sleep disorder	Kaya et al., 2020	26	Alberti et al., 2003
Paget's disease	Wheeler et al., 1995	498	Numan et al., 2015
Periodontitis	Schertel Cassiano et al., 2023	50	Cecoro et al., 2020
Polycystic ovary syndrome	Koseoglu et al., 2016	55	Dabravolski et al., 2021
Premature menopause	Lee et al., 2019	104	Bertone-Johnson et al., 2019
Psoriasis	Zhong et al., 2023	10,918	Kommoss et al., 2023
Sarcopenia	Harita et al., 2019	141	Dalle et al., 2017
Spondyloarthritis	Yalcinkaya et al., 2019	50	Sieper and Poddubnyy, 2017
Systemic lupus erythematosus	Schoenfeld et al., 2009	100	Frangou et al., 2019
Systemic sclerosis	Amital et al., 2014	65	Volkmann et al., 2023
Testosterone deficiency	Kirgezen et al., 2021	70	Mohamad et al., 2019
Ultra-processed diet	Stevenson et al., 2020	222	Tristan Asensi et al., 2023
Vitamin B12 deficiency	Derin et al., 2016	63	Al-Daghri et al., 2016
Vitamin D deficiency	Bigman, 2020	2,216	Yin and Agrawal, 2014
Wegener's granulomatosis	Laudien et al., 2009	76	Hajj-Ali et al., 2015

NS. not specified.

**Table 3:** Congenital/hereditary disorder, the reference for accompanying olfactory dysfunction, study size of olfactory study, and reference for inflammation.

**Medical condition**	**Olfactory dysfunction**	**Olfactory study size (*N*)**	**Inflammation**
22q11 deletion syndrome	Sobin et al., 2006	62	Dou et al., 2020
Angioedema (hereditary)	Perricone et al., 2011	60	Maas and López-Lera, 2019
Bardet-Biedl syndrome	Iannaccone et al., 2005	15	Melluso et al., 2023
Cystic fibrosis	Miller et al., 2023	76	McElvaney et al., 2019
Down syndrome	Cecchini et al., 2016	56	Huggard et al., 2020
Fragile X syndrome	Juncos et al., 2012	83	Van Dijck et al., 2020
Friedreich ataxia	Connelly et al., 2002	35	Apolloni et al., 2022
Gaucher disease	McNeill et al., 2012	60	Francelle and Mazzulli, 2022
Neurofibromatosis type 1	Speth et al., 2023	26	Liao et al., 2018
Niemann-Pick	Mishra et al., 2016	2	Han et al., 2023
Retinitis pigmentosa	Charbel Issa et al., 2018	9	Zhao et al., 2022
Usher syndrome	Ribeiro et al., 2016	130	Castiglione and Möller, 2022
Wilson's disease	Chen L. et al., 2021	50	Wu et al., 2019
Wolfram syndrome	Alfaro et al., 2020	40	Panfili et al., 2021

Many of the associations between olfactory loss and medical conditions are supported by a single study. However, there are several conditions that have been studied extensively and there is strong support that has been reviewed for the relationship between these conditions and olfactory dysfunction: COVID-19 (Las Casas Lima et al., 2022), Alzheimer's disease (McLaren and Kawaja, 2024), Parkinson's disease (Bagherieh et al., 2023), depression (Kohli et al., 2016), and rhinitis (Ahmed and Rowan, 2020).

#### 1.1.2 Olfactory dysfunction occurs early in the development of some medical conditions

To show that olfactory loss increases the risk of developing symptoms of medical conditions, one would need to show that olfactory dysfunction arises before the medical condition. The relevant experiments are quite difficult to do because one must evaluate the olfactory ability of many individuals and then follow them for years to determine whether poor olfactory ability precedes the medical condition. Despite the challenge, several such studies have been conducted. Olfactory loss appears well before any other Parkinson's symptoms (Walker et al., 2021), and similarly, an early symptom of Alzheimer's disease is the loss of olfaction (Serby et al., 1991), with the first part of the brain to deteriorate in that disease being the olfactory pathway (Peters et al., 2003). Schizophrenia is associated with olfactory dysfunction and such dysfunction can be seen in youths who eventually develop schizophrenia (Kamath et al., 2012). Olfactory loss also precedes depression (Kamath et al., 2024), major cardiac events (Chamberlin et al., 2024), and multiple sclerosis (Constantinescu et al., 1994); olfactory dysfunction therefore appears to be a prodromal symptom of these conditions.

#### 1.1.3 Olfactory dysfunction prospectively predicts cognitive loss and all-cause mortality

In men, significant correlations are found in measurements of olfactory thresholds and language index score, along with correlations with executive function. On the other hand, women had correlations for olfactory discrimination and olfactory identification with a visuospatial index score (Masala et al., 2024). In young adults, olfactory ability is correlated with cognitive performance as assessed by verbal fluency, word list learning, word list recall, and the Trail Making Tests, even when the outcomes were adjusted for age, sex, education, and depression symptoms (Yahiaoui-Doktor et al., 2019). Challakere Ramaswamy and Schofield (2022) reviewed 54 studies and found a variety of cognitive abilities that correlated with olfactory ability, including: impulsivity, processing speed, inhibitory control, verbal fluency, working memory, mental flexibility, decision-making, visuospatial processing, planning, and executive function.

If olfactory loss has a causal relationship with at least some medical conditions, one might expect that the loss of olfaction would predict the incidence of those conditions. Indeed, one can predict the probability that older adults will later develop mild cognitive impairment (MCI) based on their olfactory ability (Wheeler and Murphy, 2021). Furthermore, of those individuals who have MCI, one can predict which individuals will develop Alzheimer's disease, as well as which individuals will descend rapidly into their dementia, based on their olfactory ability (Wheeler and Murphy, 2021). Parkinson's patients have both a loss of olfactory function and a loss of executive function (Solla et al., 2023). There are now a number of large prospective cohort studies showing that olfactory ability is a strong predictive factor for all-cause mortality up to 17 years later (Wilson et al., 2011; Gopinath et al., 2012; Pinto et al., 2014; Devanand et al., 2015; Ekström et al., 2017; Schubert et al., 2017; Fuller-Thomson and Fuller-Thomson, 2019; Kamath and Leff, 2019; Liu et al., 2019; Choi et al., 2021; Pinto, 2021; Xiao et al., 2021; Pang N. Y. et al., 2022), with higher accuracy than predictions based on heart disease (Pinto et al., 2014).

### 1.2 Mechanisms linking olfactory loss and medical conditions: inflammation, neuroanatomy, environmental stressors

#### 1.2.1 Mechanism for triggering olfactory system damage

There are several possibilities for the mechanism underlying the many associations between olfaction and disease. One possibility is that there is a common mechanism that affects both the olfactory system and various neurological and somatic targets. Another possibility is that the neurological and somatic conditions produce something that degrades the olfactory system. A third possibility is that the olfactory system produces something that puts the brain and the body at risk either for contracting diseases or for expressing the symptoms of those diseases. One common product of disease is inflammation, and there is a strong relationship between olfactory dysfunction and elevated inflammation. As can be seen in Tables 1–3, at least 139 conditions that are associated with olfactory loss are also associated with increased inflammatory responses. These conditions have been subdivided into three separate categories: neurological, somatic, and congenital/hereditary conditions (Tables 1–3, respectively). Although the conditions could have been further subdivided into many other more specific categories, and some of the conditions may fall under two different categories, for simplicity, each medical condition was included in only one of the three categories.

#### 1.2.2 Inflammation may be causing the olfactory dysfunction

Perhaps the olfactory system is particularly sensitive to inflammation that reaches it either from other parts of the brain or through the peripheral bloodstream. Alternatively, inflammation in the olfactory system may be triggered by agents that enter through the nose, such as air pollution (Ajmani et al., 2017) or unpleasant odors (Anja Juran et al., 2022). In addition, olfactory dysfunction associated with SARS-CoV-2 (COVID-19) infection is thought to be mediated in part via inflammation (Chang et al., 2024). The olfactory system may be uniquely sensitive to damage inflicted by other sources of inflammation (brain or body) that arise from various diseases because it is already sustaining high levels of inflammation from exposure to volatile agents from the air.

Poor ability to sniff contributes to the olfactory dysfunction of Parkinson's patients (Sobel et al., 2001). The ability to sniff predicted performance on olfactory tasks and increasing sniff vigor improved olfactory ability. The problems with sniffing may be due to increased inflammation that may prevent the respiratory system from compensating for the olfactory dysfunction (Huxtable et al., 2011).

Murphy et al. (2024) found that the efficacy of olfactory training for those individuals who had lost their olfactory ability after a COVID-19 infection was quite variable, with large differences in outcomes for different age groups. They surveyed more than 5,500 patients who had olfactory dysfunction following COVID-19 and compared the efficacy of various treatments including steroids and olfactory training. They found that nasal steroid use, given to reduce inflammation, was most effective for those 25–39 years old, with their effectiveness at about 25%, while oral steroid use was most effective for 18–24-year-olds, nearing 50%. Nasal steroids were most effective for treating hyposmia (poor olfactory ability), while oral steroids were most effective for phantosmia (imagined odors). Olfactory training was most effective for 18–24-year-olds, with effectiveness nearing 50%, while 40–60-year-olds had very poor effectiveness scores. Olfactory training was most effective for hyposmia.

Interestingly, several scents have been shown to have anti-inflammatory action in animal models, including: eucalyptol (Juergens et al., 2003), 1,8-cineol (Pries et al., 2023), lavender (Ueno-Iio et al., 2014), ginger (Aimbire et al., 2007), carvacrol (Alavinezhad et al., 2018), Shirazi thyme (Alavinezhad et al., 2017), farnesol (Ku and Lin, 2016), thymoquinone (El Gazzar et al., 2006, thymol (Gholijani et al., 2016), limonene (Hirota et al., 2012), citronellol (Pina et al., 2019), α-terpineol (Pina et al., 2019), Mentha piperita (Hudz et al., 2023), and mango (Rivera et al., 2011; see Ramsey et al., 2020 and Gandhi et al., 2020 for reviews).

The links between olfaction and inflammation seem also to be mediated by diet. Transgenic mice with high levels of the apolipoprotein E gene APOE4 (a risk factor for Alzheimer's disease) and given a diet with low docosahexaenoic acid (an omega-3 fatty acid) had olfactory loss and memory loss along with an increase in IBA-1, an inflammatory factor, in the olfactory bulb. The mice given a diet high in docosahexaenoic acid experienced no olfactory loss, cognitive loss, or elevated inflammation (González et al., 2023). Humans who have a diet low in monosaturated and polyunsaturated fats have an increased risk of both cognitive loss and olfactory loss (Vohra et al., 2023).

Although the list of conditions in which olfactory loss and inflammation co-occur is long, there do exist medical conditions that involve olfactory loss, without reports of inflammation. One example is Kallmann syndrome, in which olfactory bulb development is disordered. Individuals with this condition have olfactory loss as well as deterioration in various brain areas, but it is unclear whether the neurological differences arise from olfactory dysfunction or from the other aspects of the syndrome (Manara et al., 2014; Ottaviano et al., 2015). It certainly is possible that this condition involves an increase in inflammation, even though no one has reported it.

#### 1.2.3 Olfactory loss results in damage to brain regions central to memory function

Given the predictive nature of olfactory loss for memory impairment in dementia, the question arises as to how olfactory loss could play a role in memory loss specifically. In fact, the olfactory system is anatomically unique among the senses, in that it has a “superhighway” that bypasses the thalamus and projects directly to regions of the brain involved in memory processing (Gottfried, 2006). Multiple studies now show that loss of olfaction is associated with deterioration of several brain regions (Bitter et al., 2010a,b; Eckert et al., 2024; Han et al., 2023; Kovalová et al., 2024; Peter et al., 2023; Seubert et al., 2020; Whitcroft et al., 2023; Yao et al., 2018), including the regions of the brain integral to memory acquisition and processing. While the deterioration of brain areas may be due to olfactory loss, it is also possible that the factor that produced the olfactory dysfunction also produced the damage in the other brain areas.

#### 1.2.4 Environmental challenges compromise both olfaction and memory

Having identified inflammation as a possible global mediating factor in the links between olfactory loss and medical conditions and mortality, as well as neuroanatomical factors creating a tighter fit between olfactory loss and memory loss specifically, we can proceed to ask whether specific life experiences may activate such connections. There are indeed experiences that are known to cause both loss of olfactory ability and loss of memory, as well as the more diffuse impairments often referred to as “brain fog”. These include: smoking (Ajmani et al., 2017; Lewis et al., 2021), air pollution (Calderón-Garcidueñas and Ayala, 2022; Wang X. et al., 2021), a wide range of medications (Schiffman, 2018; Chavant et al., 2011), stress (Hoenen et al., 2017; Shields et al., 2017), childhood maltreatment (Maier et al., 2020; O'Shea et al., 2021), illiteracy (Dong et al., 2021; Arce Rentería et al., 2019), menopause (Lee et al., 2019; Maki, 2015), toxins (Upadhyay and Holbrook, 2004; Guan et al., 2022), alcoholism (Maurage et al., 2014; Pitel et al., 2014), respiratory infections (Potter et al., 2020; Matsui et al., 2003), nasal passage blockage (Mohamed et al., 2019; Arslan et al., 2018), head trauma (Lötsch et al., 2016; McInnes et al., 2017), highly processed food (Makhlouf et al., 2024; Gomes Gonçalves et al., 2023), and COVID-19 (Doty, 2022).

In one longitudinal study (Douaud et al., 2022), imaging was used to examine the effects of COVID-19 on the brain for individuals who had contracted a mild case of COVID-19 during the time between two brain scans. The second scan was completed approximately 141 days after testing positive for COVID-19, with an average time of 3 years between scans. Comparisons were made with brain scans from individuals who had not tested positive between scans. In the group who had contracted COVID-19, the researchers found significant damage in the regions of the brain involved in olfaction and memory, including the anterior cingulate cortex, orbitofrontal cortex, ventral striatum, amygdala, hippocampus, and parahippocampal gyrus, and the extent of olfactory loss predicted the extent of the brain damage (Campabadal et al., 2023). These individuals also continued to experience cognitive loss.

#### 1.2.5 Olfactory dysfunction and cognitive loss

Compared to our ancestors, most humans in the affluent world experience a narrower range of evolutionarily relevant odors. In addition, people typically have experiences that damage their olfactory system: air pollution, stress, toxins, anatomical blockage, smoking, various medications, adverse childhood experiences, menopause, and even chronic sinusitis, all of which also trigger memory loss (Eimer and Vassar, 2013). As people age, the deterioration of their olfactory ability accompanies the deterioration of their cognitive ability (Leon and Woo, 2022; Doty et al., 1984), perhaps because olfactory loss results in a significant loss of both gray matter and white matter in the cognitive areas of human brains (Schaie et al., 2004; Bitter et al., 2010a,b).

##### 1.2.5.1 Olfactory loss accompanies dementia

Olfactory dysfunction predicts cognitive dysfunction in humans (Schubert et al., 2008) and the loss of olfactory function precedes or parallels the initiation of a wide variety of cognitive disorders such as: AD, MCI, Parkinson's disease, Lewy body dementia, frontotemporal dementia, Creutzfeldt-Jakob disease, alcoholism, and schizophrenia (Wang Q. et al., 2021; Conti et al., 2013; Adams et al., 2018; Ponsen et al., 2004; Birte-Antina et al., 2018).

##### 1.2.5.2 COVID-19 links olfactory loss and dementia

COVID-19 typically produces olfactory loss, and comparisons of MRI scans from individuals both pre-infection and post-infection have revealed neural deterioration that resembles a decade of aging in the cognitive brain regions that receive olfactory-system projections, along with damage to those areas involved in olfaction (Kollndorfer et al., 2015; Segura et al., 2013). Kay (2022) made the case that COVID-19 infections that produce olfactory loss may foster the cognitive loss that is seen in Alzheimer's disease. In fact, Wang et al. (2022) did a retrospective study of 6,245,282 older adults and showed that people with COVID-19 were at significantly increased risk for new diagnosis of Alzheimer's disease within 360 days after the initial COVID-19 diagnosis. Rahmati et al. (2023) went on to do a meta-analysis of twelve studies tracking over 33 million individuals who either had contracted COVID-19 or did not contract the virus. The pooled analyses showed a significant association between COVID-19 infection and subsequent increased risk for new-onset Alzheimer's disease. Given the remarkable number of physiological systems that were affected by the disease (Nasserie et al., 2021), there is no reason to believe that the olfactory loss was the sole factor in increasing the risk of Alzheimer's, but it may be that the loss of olfaction contributed to the degradation of regions in the brain integral to normal memory functioning, as mentioned previously (Kovalová et al., 2024).

### 1.3 Efficacy of olfactory enrichment

#### 1.3.1 Olfactory enrichment improves symptoms of cognitive impairment

Shi et al. (2023) reviewed a number of studies examining the effects of exposure to essential oils and found a wide range of benefits to the brain and behavior. The benefits included normalizing neurotransmitter levels, decreasing inflammatory factors, decreasing oxidation, increasing neuroprotective factors, improving memory, decreasing neuronal loss, and suppressing beta amyloid levels.

#### 1.3.2 Olfactory enrichment results in memory benefits for healthy adults

From a preventive perspective, about 20 studies have now been performed showing that increasing olfactory stimulation can improve memory (Vance et al., 2024).

For example, olfactory enrichment improves cognition in older adults. Birte-Antina et al. (2018) provided olfactory enrichment with 4 essential-oil odorants twice a day for 5 months, while controls solved daily Sudoku puzzles. The olfactory-enriched group had a significant improvement of olfactory function, improved verbal function, and decreased depression symptoms. Oleszkiewicz et al. (2022) exposed 68 older adults either to 9 odorants twice a day or to no new odorants for 3–6 months, and found the enriched olfactory experience produced improvements in cognitive abilities, dementia status, and olfactory function relative to controls. Specifically, the Montreal Cognitive Assessment revealed a significant improvement in the olfactory-enriched group relative to controls, and the AD8 Dementia Screening Interview showed that enriched participants had no increase in dementia symptoms over the trial period, while control participants had an increase in symptoms.

Increased complexity of olfactory enrichment also improves dementia symptoms. Cha et al. (2022) exposed 34 older adults with dementia to 40 odorants twice a day for 15 days. The control group consisting of 31 individuals with dementia received no such stimulation. There were no initial differences between groups, and all had a Mini-Mental Status Examination score of at least 10. The results were remarkable, as the olfactory-enriched group showed highly significant improvements in memory, olfactory identification, depression symptoms, attention, and language skills. Olfactory-enriched individuals improved their olfactory identification, while controls did not. The Verbal Fluency Test also showed significant improvements for the enriched group relative to the controls. Similarly, the Boston Naming Test revealed a significant improvement in the enriched subjects relative to controls. The Word-List Memory Test, the Word-List Recall Test, the Word List Recognition Test, and the Geriatric Depression Scale all improved in the enriched group relative to controls.

Lin and Li (2022) exposed older adults with mild-to-moderate dementia to 15 essential oils/essences twice a week for 30-min sessions over a 12-week randomized clinical trial. Participants in the olfactory enrichment group also were asked to relate each scent to a matching photo of the scent source. The olfactory enrichment group showed significant cognitive improvement on the Loewenstein Occupational Therapy Cognitive Assessment-Geriatric test. In addition, olfactory enrichment prevented the increase in plasma beta amyloid seen in the control group.

In an effort to minimize burden and increase compliance, we tested the idea that we could get enhanced neural and cognitive outcomes after even minimal olfactory enrichment at night (Woo et al., 2023). The limitations of the available diffusion devices at the time forced us to use this minimal level of olfactory enrichment. Therefore, we gave olfactory enrichment or control exposures to older adults (60–85 years old) for 2 h every night for 6 months, using a single odorant each night, rotating through seven scents a week (Woo et al., 2023). There were statistically significant differences between enriched and control older adults in their cognitive ability using the Rey Auditory Verbal Learning Test, with enriched individuals scoring 226% better than controls. We also found a statistically significant change in mean diffusivity in the uncinate fasciculus of the enriched group compared to controls.

### 1.4 Mechanisms of olfactory enrichment: inflammation, neuroanatomy, and cognitive reserve

#### 1.4.1 Reduction of inflammation may be the mechanism by which olfactory enrichment improves neurological symptoms

A range of correlational and causal relationships connect inflammation with olfactory loss. Olfactory loss is associated with an increase in Interleukin-6 (IL-6), which increases both inflammation and the maturation of B cells (Henkin et al., 2013) and is also correlated with an increase in C-reactive protein, which increases in the presence of inflammation as indicated by IL-6 (Ekström et al., 2021). Chronic inflammation is associated with olfactory dysfunction (LaFever and Imamura, 2022). As noted earlier, a proinflammatory diet for older adults with low levels of polyunsaturated fatty acids and monosaturated fatty acids is associated with elevated inflammation, olfactory dysfunction, and cognitive decline (Vohra et al., 2023). Moreover, such a diet increases the risk of dementia (Simopoulos, 2002). The association between olfactory dysfunction and frailty varies with the level of inflammation, as measured by circulating levels of the pro-inflammatory cytokine IL-6 (Laudisio et al., 2019). Hahad et al. (2020) found that inflammation mediated the loss of cognition in those exposed to high levels of pollution.

Turning to causal links, unpleasant odors activate the inflammatory response by increasing tumor necrosis factor alpha (TNFα) and decreasing secretory immunoglobulin A (slgA) in saliva (Anja Juran et al., 2022). Imamura and Hasegawa-Ishii (2016) found that toxins can activate the immune response in the olfactory mucosa. Conversely, smelling pleasant odors suppresses immune activity, and more strikingly, even the act of imagining pleasant odors suppresses the immune response, specifically circulating interleukin-2 (IL-2; Matsunaga et al., 2013; Shibata et al., 1991). Casares et al. (2023) found that 6 months of exposure to menthol odor improved both the memory of young mice and the memory of mice that were modified to model Alzheimer's disease. This odor exposure also suppressed inflammation (IL-1β; Casares et al., 2023). Equally, pharmaceutical suppression of inflammation in those mice improved their memory (Casares et al., 2023).

The suppression of the inflammatory response may therefore underlie the finding that olfactory enrichment can improve memory (Cha et al., 2022; Woo et al., 2023). In addition, olfactory enrichment may improve symptoms of other neurological conditions through a similar mechanism.

#### 1.4.2 Olfactory enrichment creates functional and structural changes in the brain

Increased olfactory stimulation, as experienced daily by master perfumers and sommeliers, who sample many odors each day for months and years, results in increased volume of brain regions that receive olfactory projections (Royet et al., 2013; Filiz et al., 2022). A longitudinal study showed that after a year and a half of olfactory training, sommeliers in training, who sampled dozens of odors every day for months to be able to identify those odors in fine wines, increased the thickness of their entorhinal cortex, a brain region critical for memory formation and consolidation (Filiz et al., 2022; Takehara-Nishiuchi, 2014). This structural change may well have functional benefits. Daily olfactory training for 6 weeks resulted in improved olfactory functioning as well as increased cortical thickness of olfactory processing regions of the brain (Al Aïn et al., 2019), and multiple scents presented daily improved cognition in both adults and older adults (Oleszkiewicz et al., 2021, 2022). Additionally, reversal of some medical issues, such as removing an anatomical blockade in the nasal passages, can result in improved cognition and attention, as measured using neuropsychological assessments and event-related auditory evoked potentials (P300; Arslan et al., 2018). In the memory study with healthy older adults described above (Woo et al., 2023), the enriched group that showed improvement in memory performance also had a statistically significant change in mean diffusivity in the uncinate fasciculus, a brain pathway involved with maintaining cognitive processes.

#### 1.4.3 Electrical stimulation of the olfactory system

One mechanism by which olfactory enrichment may be working is by stimulating specific brain areas. Beta amyloid (Aβ) is elevated in Alzheimer's disease (Pignataro and Middei, 2017). In a rat model of Alzheimer's disease, electrical stimulation of the olfactory bulb reversed the accumulation of beta amyloid (Aβ) plaque formation in the prefrontal cortex, the entorhinal cortex, the dorsal hippocampus, and the ventral hippocampus. It also blocked the impairments in working memory in these rats (Salimi et al., 2024). In addition, electrical stimulation of the olfactory bulb also increased functional connectivity in the brain during a working memory task. It should be noted that transethmoid electrical stimulation of the human olfactory bulb induced olfactory perceptions (Holbrook et al., 2019). Olfactory enrichment may therefore act to stimulate the areas to which the olfactory input projects (Gottfried, 2006). Conversely, intrabulbar injections of Aβ in rats decreased olfactory function, a phenomenon that was more easily triggered in older rats (Alvarado-Martínez et al., 2013).

#### 1.4.4 Making a distinction between contracting a disease vs. experiencing symptoms of a disease

It is important in a discussion regarding causality to consider whether something can differentially change the risk of *contracting* a disease or the risk of *experiencing the symptoms* of the disorder. This distinction may be important for our understanding of the relationships between olfaction, cognition, and disease. Typically, the symptoms of the disease accompany the disease itself, but there are exceptions. Some people who contracted the COVID-19 virus, for instance, did not show any symptoms of the disease (Rasmussen and Popescu, 2021). In the phenomenon called cognitive reserve, an individual can develop the neuropathology of Alzheimer's disease, indicating that they had contracted the disease, but show none of the symptoms of severe memory loss (Stern, 2012).

#### 1.4.5 Olfactory ability and cognitive reserve

In mice, long-term olfactory enrichment improves olfactory ability, and it also improves learning and memory for tasks that do not involve odors (Terrier et al., 2024). This effect may represent a form of cognitive reserve in mice, here mediated by an increase in noradrenergic innervation and resulting in the remodeling of brain connectivity in older mice. These data suggest a causal association between olfactory enrichment and cognition. In humans, odor threshold correlates with a measure of cognitive reserve that involves education, while odor discrimination ability correlates with career experiences and leisure experiences. Women had significant correlations between odor threshold, discrimination and identification, and leisure experiences, while men had a significant association between odor threshold and educational experiences (Masala et al., 2023).

#### 1.4.6 Olfactory enrichment may induce cognitive reserve in humans

Cognitive reserve in humans comes from leading a life filled with environmental enrichment, with a high level of education, a cognitively engaging career, and a high level of socializing (Stern, 2012). Conversely, illiterate individuals have the highest probability of developing Alzheimer's disease (Dong et al., 2021), and they have little of the enrichment that seems to protect those with cognitive reserve (Brucki, 2010). Perhaps the uniquely direct connections of the olfactory system to the regions of the brain that are critical for memory functioning allow the olfactory system to rapidly induce what may be called cognitive reserve in humans.

## 2 Discussion

There is reason to believe that the relationship between olfactory loss and medical conditions may be more than coincidental. First, there are many instances where both are present, with at least 139 medical conditions showing associations with olfactory dysfunction. Second, olfactory loss precedes the expression of the medical condition, raising the possibility that olfactory loss makes the brain or body vulnerable to expressing the symptoms of these medical conditions. Third, olfactory loss prospectively predicts both memory loss and all-cause mortality.

Inflammation could be a key mechanism underlying a causal relationship between olfaction and memory; neuroanatomical and environmental factors also play a role. While the causal arrow may go either way, it is possible that for some conditions, it is the olfactory loss that raises the risk of expressing the symptoms of those conditions.

If olfactory loss increases the risk of either developing these medical conditions or having the symptoms of the conditions, then it may be possible to prevent the onset of symptoms from these conditions. Studies show that olfactory enrichment improves memory performance in healthy adults and there are even greater improvements found for adults with dementia. These benefits may be mediated via reduction of inflammation.

A suggestive notion underlying many of these observations is that neuropathology is not always symptomatic, thanks to phenomena such as cognitive reserve. For instance, people with cognitive reserve have the neuropathology of Alzheimer's disease, but they don't have the memory-loss symptoms. The olfactory system may be involved in generating protective cognitive reserve especially for memory-related conditions. More widely, since pleasant scents can decrease harmful inflammation, it seems possible that olfactory enrichment may reduce the symptoms of other medical conditions.

Future directions for research in this area would include simultaneously studying both olfaction and inflammation in specific medical conditions, studying more conditions in individuals who have olfactory dysfunction, and studying these variables over time. It also would be interesting to block inflammation in specific medical conditions to determine the effects on olfaction.

## Data Availability

The original contributions presented in the study are included in the article/supplementary material, further inquiries can be directed to the corresponding author.
